# Target delineation workflow and outcomes of stereotactic cardiac radioablation

**DOI:** 10.1016/j.hroo.2025.06.009

**Published:** 2025-06-19

**Authors:** Muhammad R. Afzal, Emile G. Daoud, Mahmoud Gomaa, Toshimasa Okabe, Salvatore J. Savona, Natee Sirinvaravong, Matthew Tong, Mahmoud Houmsse, Ralph S. Augostini, Steven J. Kalbfleisch, Eric Miller, Evan Thomas, Terence M. Williams, Michael Weldon, John D. Hummel, Jeremy Brownstein

**Affiliations:** 1 Section of Electrophysiology, Division of Cardiovascular Medicine, The Ohio State University Wexner Medical Center, Columbus, Ohio; 2 Section of Electrophysiology, TriHealth Heart & Vascular Institute, Cincinnati, Ohio; 3 Section of Electrophysiology, Heart and Vascular Services, Swedish Medical Center, Seattle, Washington; 4 Department of Radiation Oncology, James Cancer Center, The Ohio State University Wexner Medical Center, Columbus, Ohio; 5 Department of Radiation Oncology, City of Hope National Medical Center, Duarte, California

**Keywords:** Ventricular tachycardia, Radiation ablation, Cardiac imaging, Ventricular scar, Electroanatomic mapping, Arrhythmias

## Abstract

**Background:**

Stereotactic body radiation therapy (SBRT) is an emerging modality for the treatment of ventricular tachycardia (VT). The workflow for delineation of the SBRT target is evolving.

**Objective:**

This project describes the procedural workflow and outcomes of SBRT for VT.

**Methods:**

The primary indication for SBRT was recurrent VT despite maximal contemporary treatment. Target delineation for SBRT involved combining imaging and electrophysiological data. VT burden, defined as the number of sustained VT episodes per month, was compared as the primary outcome. Secondary outcomes assessed included reduction of antitachycardia pacing and defibrillator shock episodes and reduction in the number of antiarrhythmic drugs per patient during follow-up.

**Results:**

Workup for VT target delineation and radiation delivery was conducted in 25 patients receiving 27 SBRT procedures. VT management prior to SBRT consideration included ≥2 catheter ablations in 22 (88%) and surgical sympathectomy in 7 patients (28%). Of the 27 performed cases, SBRT target delineation incorporated electrocardiogram of clinical VT in 16 (59%), at least 2 noninvasive imaging modalities to assess scar in 24 (89%), and invasive electroanatomic mapping in 25 (93%). Among 16 patients with a complete 6-month follow-up, the reduction of VT burden per month was 81% (*P* < .05). Reduction in antitachycardia pacing and defibrillator shocks per month was 86% and 98%, respectively (*P* < .05). The number of patients on ≥2 antiarrhythmic drugs decreased from 69% to 0% (*P* < .01). One patient developed diaphragmatic paralysis after SBRT.

**Conclusion:**

In patients with recurrent VT despite maximal contemporary antiarrhythmic therapies, SBRT offers a safe alternative once the target is adequately delineated by combining imaging and electrophysiological data.

**Key Findings**

▪ Multidisciplinary workflow is essential: stereotactic body radiation therapy (SBRT) target delineation was achieved through the integration of imaging, electroanatomic mapping, and electrophysiological data, requiring collaboration among electrophysiologists and radiation oncologists.
▪ Significant reduction in ventricular tachycardia (VT) burden: SBRT resulted in a significant reduction in sustained VT burden, as well as implantable cardioverter-defibrillator–delivered antitachycardia pacing and shock therapies, among patients with refractory VT who survived to the 6-month follow-up without requiring cardiac transplantation.
▪ SBRT is feasible and well tolerated: most SBRT sessions were performed in the outpatient setting, with good tolerability and no acute procedural complications.
▪ Radiation dose management is critical: selective underdosing and strict dose constraints on surrounding structures are necessary to limit the risk of radiation toxicity. This reflects the importance of individualized radiation planning.

## Introduction

Despite progress in the understanding of the mechanism of ventricular tachycardia (VT), the management options remain limited. Widely available strategies for VT management include antiarrhythmic drugs (AADs) and catheter ablation (CA). CA is often facilitated by altering irrigation fluid, unipolar/true bipolar ablation, intramyocardial and epicardial radiofrequency, and use of ethanol ablation.^1^^,^^2^ Modification of sympathetic innervation of the heart via percutaneous or surgical approach is also beneficial in some patients with refractory VT.^3^

For patients in whom ablation therapy cannot be achieved with a catheter-based approach, stereotactic body radiation therapy (SBRT) has been proposed as an additional technique.^4^, ^5^, ^6^, ^7^ SBRT can be viewed as a complimentary strategy to radiofrequency in patients where an adequate ablation lesion could not be delivered owing to limitations of current CA technology. In addition, the potential noninvasive nature of SBRT makes it an attractive option for patients with multiple comorbidities who are considered high risk of CA.^8^

Studies to date regarding SBRT have described varied methods to delineate the SBRT treatment zone; thus, a consistent and successful workflow has not been identified.^5^^,^^7^^,^^9^ In part, the absence of a standardized SBRT workflow has limited the broader utilization of SBRT.^10^ This study’s objective is to describe the procedural workflow and report safety and efficacy outcomes in patients undergoing SBRT.

## Methods

### Patient population and inclusion criteria

Eligible patients were enrolled in a prospective registry that included all patients who had refractory VT despite at least 2 AAD trials and either failed or were ineligible for at least 1 CA. Patients who were ineligible for CA had the presence of mechanical aortic and mitral valves, left ventricular (LV) thrombus, or LV aneurysm precluding safe catheter manipulation. The study was approved by the Institutional Review Board of The Ohio State University, and all patients gave informed consent. The research was conducted in accordance with the principles of the Declaration of Helsinki.

### Noninvasive SBRT workup

**Figure 1:**
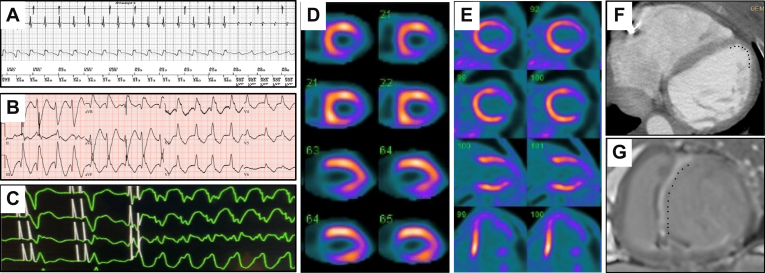
Representative noninvasive electrophysiological and imaging data to delineate target. **A:** Device interrogation revealing electrogram for a monomorphic ventricular tachycardia (VT) at 162 beats (cycle length = 370 ms). **B:** Twelve-lead electrocardiogram revealing VT with a right bundle inferior axis morphology with positive precordial concordance indicating a basal anterior left ventricular origin. **C:** Telemetry strip during inpatient monitoring of the patient revealing VT morphology as right bundle, superior axis. **D:** Single photon emission computed tomography and **E:** Positron emission tomography scan showing a lack of metabolic activity in the mid- to lateral apical wall. **F:** Cardiac computed tomography with contrast showing midanterior wall thinning consistent with scar from previous anterior wall myocardial infarction. **G:** Cardiac magnetic resonance imaging showing delayed gadolinium enhancement consistent with anteroseptal scar.

Noninvasive electrophysiological (EP) characteristics of VT were obtained from device interrogations (electrograms revealing rate of VT), telemetry data from ambulatory or inpatient monitoring, or 12-lead electrocardiograms (EKGs) of clinical VT if available. Target localization based on EKG data was performed as described previously.^11^ Noninvasive imaging data included transthoracic echocardiogram showing ventricular wall thinning and/or hypokinesis or akinesis, cardiac computed tomography (CT) with contrast to show myocardial thinning, magnetic resonance imaging (MRI) showing late gadolinium enhancement, single-photon emission CT showing a lack of radiotracer uptake, and positron emission tomography showing a lack of metabolic activity as a surrogate for ventricular scar (Figure 1). Noninvasive EP and imaging workup discerned areas of high probability for VT based upon EKG criteria and scar distribution.

### Invasive SBRT workup

**Figure 2:**
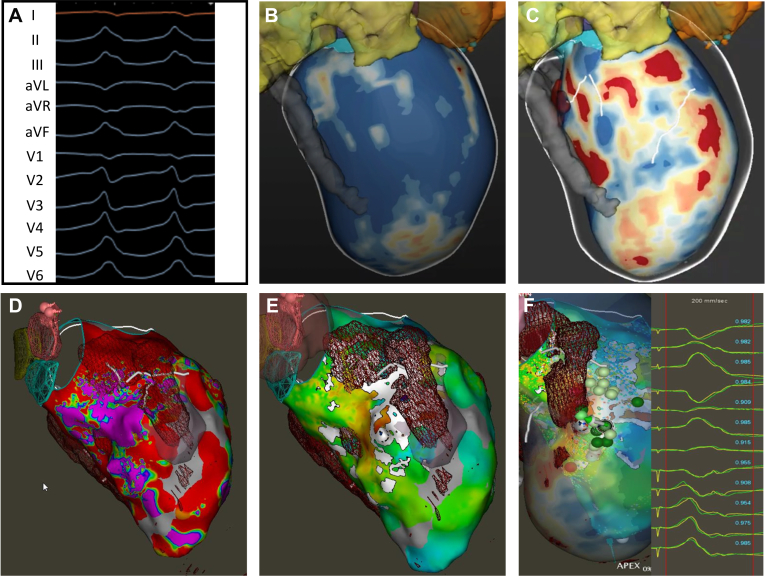
Target delineation in a case with agreement of imaging-based scar and electrophysiological data. **A:** Ventricular tachycardia (left bundle, inferior axis with V2 precordial transition suggestive of left ventricular outflow tract origin) induced during noninvasive electrophysiological study using s3 protocol. **B and C:** Cardiac magnetic resonance image processing using ADAS software showing healthy tissue in the epicardial aspect (*blue color*) and scar (*red*) in the endocardial aspect. The scar-mediated ventricular tachycardia channel is identified as a *white line* between 2 scar (*red*) areas. There is an endocardial scar in the midanteroseptal area. **D:** Endocardial bipolar voltage with presence of scar (*red*) in the basal to midanteroseptal area. *Purple area* identifies healthy voltage. **E:** The *brown mesh* identifies the magnetic resonance imaging–based scar, and the *white area* identifies the presence of wavefront discontinuity lines in an activation map obtained in sinus rhythm. **F:** Pace mapping at the areas of interest. The pace-map agreement of >95% labeled as *dark green* and >90% as *light green dots*.

All patients, except those in whom LV access was precluded, had invasive mapping data. Invasive EP data were either from the most recent VT ablation or from a dedicated EP study performed as part of workup for SBRT. Detailed 3-dimensional electroanatomic maps (EAMs) were generated by incorporating multiple mapping modalities. First, a high-density map of the LV was completed during sinus rhythm or ventricular pacing to identify endocardial scar, defined as low voltage at <0.5 mV. Areas of fractionation, late potentials, slow conduction, and wavefront discontinuity lines were identified as described previously.^12^ Second, detailed cardiac anatomy of ventricular chamber contour, mitral isthmus, and aortic cusps and defibrillator and coronary sinus lead were marked using intracardiac echocardiogram. The location of the conduction system was determined by identifying electrograms corresponding to the His bundle and fascicular pathways in reference to standard intracardiac landmarks such as the noncoronary cusp on the intracardiac echocardiogram. Third, programmed stimulation at 2 distinct right ventricular sites using 3 drive cycle lengths with 3 extrastimuli was performed to induce any forms of (clinical and nonclinical) ventricular arrhythmias. Once a monomorphic VT was induced, activation, entrainment, and pace mapping were performed. A pace mapping agreement of ≥85% was selected as guidance for the approximate arrhythmogenic substrate (Figure 2). When available, images from contrast-enhanced cardiac CT or MRI were imported into the EAM to correlate scars identified on CT/MRI with the EP data of the EAM. Additional scar data and scar-mediated VT channels were localized using ADAS Medical software in patients where cardiac MRI was available.^13^

### Combining noninvasive and invasive data for SBRT target delineation

**Figure 3:**
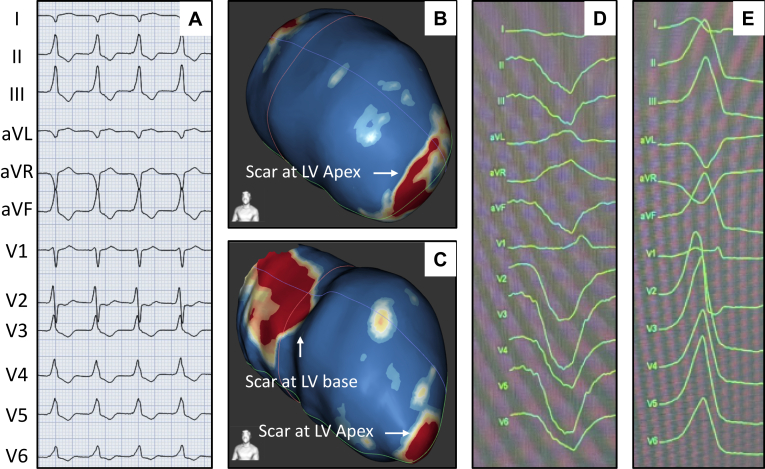
Target delineation in a case with disagreement of imaging-based scar and electrophysiological (EP) data. **A:** Spontaneous ventricular tachycardia (VT) (left bundle, inferior axis with V2 precordial transition) captured on 12-lead electrocardiogram during hospital stay. **B:** Cardiac magnetic resonance image processing using ADAS software showing healthy tissue (*blue color*) and scar (*red*) in the epicardial aspect of LV apex on a left anterior oblique view. **C:** Presence of scar (*red*) in the anterolateral area primarily in the endocardial aspect. **D:** VT induced during EP study. The morphology (left bundle, superior axis, and negative precordial concordance) suggests LV apical origin. **E:** The second VT induced during EP study (left bundle, inferior axis with V2 precordial transition) suggests basal LV origin. This VT is like a clinical VT. Therefore, the basal area was selected as the target for stereotactic body radiation therapy. LV = left ventricular.

A critical aspect of using SBRT is to identify the proper zone to target for therapy. Target delineation for SBRT is based upon correlating the noninvasive EP (EKG) and anatomic scar data with invasive EAM of the VT substrate. Using these main sources of data, a region of myocardium to treat is then identified and a manual integration of this data is performed to display the target area on the standardized 17-segment model adopted by the American Heart Association.^14^ Target delineation on the 17-segment model involved agreement of at least 2 electrophysiologists who reviewed available data such as EKGs, mapping, imaging, and previous ablation records. In case of discrepancies, findings were discussed collaboratively to reach consensus. In patients where invasive electroanatomic data could not be obtained owing to the presence of mechanical valves, the SBRT target was determined using scar imaging and EKG data. Ideally, these different strategies of identifying the likely myocardial sources of VT nicely overlap, thus confirming the region to treat with SBRT (Figure 2). In situations where imaging-based scar data and EP data (EKG, EAM) were discordant, priority was given to EP data, as described in Figure 3.

### Radiation oncology is part of SBRT target planning

Patients were secured on a Civco stereotactic frame for SBRT, with a vacuum bag, and arms up with an arm shuttle, headrest, knee sponge, foot sponge, and abdominal compression plate or belt. Abdominal compression was used to limit respiratory excursion. After immobilization, 4-dimensional (4D) contrast CT-based simulation planning was performed with 2.5 mm slice thickness. To minimize radiation dose to the stomach and ensure reproducibility of the setup, patients were asked not to eat for 4 hours prior to CT simulation or treatment. Contrast was administered during the 4D CT.

### Displaying the SBRT target on the radiation planning system

**Figure 4:**
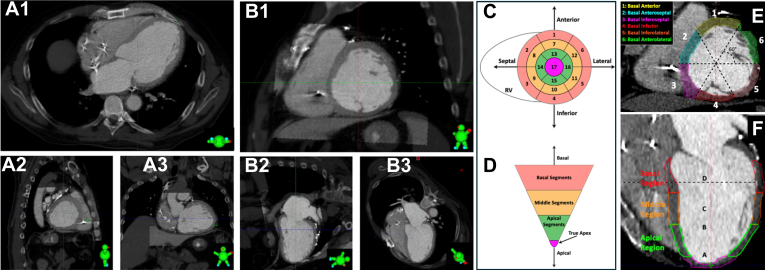
Orienting the radiation oncology computed tomography (CT) simulation study into cardiac-specific views to facilitate stereotactic body radiation therapy target delineation. **A1–A3:** Radiation oncology CT simulation study in typical views used by radiation oncologists (**A1**, axial; **A2**, sagittal; **A3**, coronal). **B1–B3:** Reorientation of radiation oncology CT simulation study for display in typical cardiac views (**B1**, short-axial view; **B2**, 2-chamber view; **B3**, 4-chamber view). **C and D:** The American Heart Association 17-segment model, depicting location of the segments within the basal, middle, or apical regions of the left ventricle in both short (**C**) and long (**D**) axis views. **E and F:** Depiction of the location of the various cardiac segments outlined on radiation oncology CT simulation study in short (**E**) and long (**F**) axis views. RV = right ventricle.

The 4D CT images were imported into the treatment planning system, and the images were fused with available diagnostic cardiac images (eg, cardiac CT, MRI, positron emission tomography/CT). The area to be treated by SBRT, called clinical target volume (CTV), was outlined (traced) on 4D CT by a multidisciplinary team consisting of a cardiac electrophysiologist, a radiation oncologist, a cardiac imaging specialist, and a physicist. First, the images on the radiation planning system were reoriented to display in typical cardiac views, as described previously (Figure 4).^15^ Next, the SBRT target that was delineated on the 17-segment model is outlined/traced on a 4D CT image on the radiation planning system. There is no automated process of transferring EAM data to the radiation planning system. This part of SBRT target delineation is prone to some subjectivity. To minimize the errors, various cardiac structures such as valve annuli, intracardiac leads, and location of conduction system were used, and EAM data were transferred to 4D CT on the radiation planning system. The estimated gross location of the conduction system was identified during contouring and deliberately excluded from the high-dose target volume when not directly involved in the arrhythmogenic substrate to minimize unintended exposure to critical conduction tissue. The CTV was anisometrically expanded to an internal target volume (ITV) that incorporates the CTV’s motion during the respiratory cycle. Finally, to address unanticipated uncertainties in setup and patient alignment, the ITV was isometrically expanded by an additional 5 mm to create the planning target volume (PTV). The CTV represented the arrhythmogenic substrate as defined by the integration of EAM, VT morphology, and scar features across imaging modalities. The ITV was generated using 4D CT or breath-hold imaging when available, to account for both cardiac and respiratory motions.

### Process to minimize collateral radiation damage

The radiation treatment plan was optimized to allow adequate coverage of the PTV with the prescription dose while keeping nearby normal tissues within safety constraints. Desired PTV coverage was 95% volume coverage with the prescription dose, although dose limits to adjacent critical normal structures often took priority. Dose limits to normal structures generally included the following: (1) spinal cord (maximum [max] point dose 1400 cGy), (2) trachea and central airways (max point dose 3000 cGy), (3) esophagus (max point dose 2400 cGy), (4) great vessels including the aorta, vena cava, pulmonary arteries, and veins (max point dose 3700 cGy), (5) rib and chest wall (maximum point dose 3000 cGy), (6) stomach (max dose 2200 cGy), (7) skin (max dose 2750 cGy), and (8) large bowel (max point dose 3000 cGy). Often, a selective underdosing of the cardiac SBRT target is done when adjacent to sensitive digestive organs (Supplemental Figure 1).

### SBRT treatment

Before treatment, patients’ vital signs were checked. Quality assurance checks were performed on the plans, and treatments were initiated as indicated. At the time of treatment, the patients were aligned using on-board cone beam CT. An additional cone beam CT was obtained halfway through the treatment to ensure that alignment remained precise. Patients were treated to a prescription dose of 2500 cGy to the target with internal boost as indicated, applied selectively in cases where imaging or EP data identified a well-defined arrhythmogenic substrate warranting focused dose escalation. Treatments used either 6 MV or 10 MV photons (flattening filter free). Throughout the treatments, a member of the EP team was always present to ensure patient stability. After the treatments were completed, vital signs were rechecked. All patients were treated at The Ohio State University Wexner Medical Center using the standardized workflow described earlier.

### Device programming

All patients with implantable cardioverter-defibrillators (ICDs) underwent device interrogation before treatment delivery. Tachycardia therapies remained on during the treatment. Cardiac rhythm was monitored during the treatment delivery.

### Primary endpoint

VT burden, defined as the number of sustained VT episodes (lasting 30 seconds or requiring antitachycardia pacing [ATP] or defibrillator shocks) per month during 3 months before and 6 months after SBRT, was compared as the primary outcome. Sustained VT was selected as the primary endpoint given that some patients had VT episodes at a rate lower than the programmed therapy rate for ATP and defibrillator shock. The reduction of ATP and defibrillator shock and the number of patients on ≥2 AADs were assessed as secondary outcomes.

### Follow-up protocol

A clinical follow-up in the electrophysiology and radiation oncology clinic was performed in all patients. In the event of symptoms suggestive of cardiac or respiratory symptoms, a transthoracic echocardiogram and noncontrast chest CT were performed. In the absence of symptoms, no routine surveillance imaging was performed.

### Statistical analysis

Continuous variables were tested for normality using the Shapiro-Wilk test. Normally distributed variables were reported as mean ± standard deviation (SD). Non-normally distributed variables were reported as median (interquartile range). Paired comparisons of the number of sustained VT, appropriate ATP, and ICD shock episodes before and after SBRT were performed using the paired *t* test. *P* < .05 was considered statistically significant. Statistical analyses were performed using Microsoft 365 Excel Software. Kaplan-Meir curve was used for time-to-event visualization (death or cardiac transplant).

## Results

### Patient population

From December 2019 to November 2024, a total of 25 patients underwent 27 individual SBRT sessions, with 2 patients receiving SBRT twice. The primary indication for SBRT was either recurrent VT refractory to AAD and CA (n = 20 [80%]) or inability to perform CA (n = 5 [20%]; mechanical valves, 2; LV thrombus, 1; LV apical aneurysm, 2). Baseline clinical characteristics are presented in Table 1. The mean age of patients was 69.4 years (SD 9.5), and most (n = 22; 88%) were men. Mean LVEF was 33.8% (SD 12.2) with most subjects (n = 23; 92%) in New York Heart Association class II/III. Most patients (n = 16; 64%) had ischemic cardiomyopathy.

**Table 1:** Baseline demographic and clinical characteristics

Variables	N = 25 patients
Age (mean ± SD)	69.4 ± 9.5
Men	22 (88%)
Body mass index (kg/m^2^) (mean ± SD)	29.9 ± 7.8
Diabetes	8 (32%)
Hypertension	22 (88%)
Atrial fibrillation	16 (64%)
COPD	3 (12%)
Chronic kidney disease	15 (60%)
LVEF (%) (mean ± SD)	33.8 ± 12.2
Cause of cardiomyopathy	
Ischemic cardiomyopathy	16 (64%)
Nonischemic cardiomyopathy	9 (32%)
Valvular	2 (8%)
Idiopathic DCM	5 (20%)
HOCM	1 (4%)
Amyloid cardiomyopathy	1 (4%)
NYHA class before SBRT	
2	10 (40%)
3	13 (52%)
4	2 (8%)

COPD = chronic obstructive pulmonary disease; DCM = dilated cardiomyopathy; HOCM = hypertrophic obstructive cardiomyopathy; LVEF = left ventricular ejection fraction; NYHA = New York Heart Association; SBRT = stereotactic body radiation therapy; SD = standard deviation.

### Previous antiarrhythmic management

VT management prior to SBRT included ≥2 antiarrhythmic medications in 21 patients (84%), ≥2 CA procedures in 22 (88%), percutaneous stellate ganglion block in 5 (20%), and bilateral cardiac sympathectomy in 7 (28%) (Supplemental Table 1). Of the 27 SBRT procedures performed, 18 (67%) were preceded by VT storm.

### Invasive and noninvasive workup for SBRT

Target delineation incorporated imaging and EP data from noninvasive and/or invasive mapping for 27 cases in 25 patients. Sixteen cases (16 of 27) had a 12-lead EKG of clinical VT. All but 1 patient had device interrogation data for rate of clinical VT, and 1 patient had a 6-lead telemetry strip showing clinical VT morphology. Among various modalities of cardiac imaging to delineate myocardial scar, all cases had at least 1 imaging study with 24 (89%) having 2 and 9 (33%) having 3 different studies (Table 2). All patients underwent invasive EP study except 2 patients, in whom mechanical mitral and aortic valves precluded LV access. Epicardial access was obtained in 5 of the 25 cases who had invasive EP study (20%). During the EP study, a monomorphic VT was induced in all cases, followed by an attempt at activation, entrainment, and pace mapping. Owing to hemodynamic instability, activation mapping could not be performed in 6 of 25 cases (24%). Adequate pace mapping (≥85% agreement) for clinical VT could be achieved in 18 of 25 cases (72%). EAM and at least 1 noninvasive imaging modality were used for SBRT in most cases (n = 20). For the 27 cases who underwent SBRT, the target location was the LV in 17 (63%) and the outflow tract in 10 (37%). Among cases with an SBRT target in the outflow tract, 9 were in the periaortic area and 1 was in the right ventricular outflow tract. Target for SBRT involved ≥2 segments on the American Heart Association 17-segment model in 26 cases (96%). Mean PTV was 131 mL (SD 34.8) (Table 2).

**Table 2:** Target localization workflow and delivery of SBRT

Preprocedure imaging	N = 27
Echocardiogram	27 (100%)
Cardiac CT	13 (48%)
Cardiac MRI	8 (30%)
SPECT scan	11 (41%)
PET scan	1 (4%)
Noninvasive EP data	N = 27
12-lead EKG of clinical VT	16 (59%)
Telemetry 6-lead EKG strips for clinical VT	1 (4%)
Device EGM for VT episode	26 (96%)
Invasive EP data	N = 25
Morphology of the predominant inducible VT	
Right bundle/superior axis	12 (48%)
Right bundle/inferior axis	5 (20%)
Left bundle/inferior axis	5 (20%)
Left bundle/superior axis	3 (12%)
Attempted invasive electroanatomic mapping	N = 25
Pace mapping ≥90%	14 (56%)
Pace mapping 85%–89%	4 (16%)
Epicardial mapping	5 (20%)
Adequate voltage map for scar characterization	20 (80%)
Activation map for induced clinical VT	19 (76%)
Unable to get adequate pace mapping	7 (28%)
Agreement of inducible VT during EP study and clinical VT	10/16 (63%)∗
Agreement between the imaging-based scar and the final SBRT target	15/27 (56%)†
Mean planning treatment volume (mL)	131.3 ± 34.8
SBRT target location	N = 27
Left ventricle	17 (63%)
Outflow tract	10 (37%)
Periaortic area	9 (33%)
RV outflow	1 (4%)
Number of targeted segments on the 17-segment model	
1	1 (4%)
2	11 (40%)
3	7 (26%)
4	7 (26%)
5	1 (4%)

CT = computed tomography; EGM = electrogram; EKG = electrocardiogram; EP **=** electrophysiological; MRI = magnetic resonance imaging; PET = positron emission tomography; RV = right ventricle; SBRT = stereotactic body radiation therapy; SPECT = single-photon emission computed tomography; VT = ventricular tachycardia.

∗ Of 16 cases with documented clinical VT in a 12-lead EKG.

† Of 27 cases who underwent SBRT and had noninvasive imaging prior to the procedure (cardiac CT, MRI, or nuclear imaging).

### Reconciliation of disagreement between noninvasive and invasive data

A total of 16 patients had a 12-lead EKG for clinical VT. All patients had inducible VT during EP study; however, inducible VT was like clinical VT in 10 cases (63%). In cases where inducible VT was different from inducible VT, clinical VT was considered as gold standard for SBRT target delineation. When the data from both noninvasive imaging and EP study were taken into consideration, the SBRT target in 11 patients was in disparate areas than suggested by noninvasive imaging. In these cases, EP study data were given priority for SBRT target delineation (Table 2).

### SBRT delivery

Notably, 25 patients underwent 27 individual SBRT sessions. Overall, 23 SBRT treatments (85%) were performed on outpatients whereas 4 treatments (15%) were performed while patients were admitted in the hospital. Among the patients who were treated as inpatient, 1 patient was intubated and sedated during the SBRT session with continuous amiodarone and lidocaine infusion; 26 cases (97%) were treated with a single fraction of 2500 cGy whereas 1 patient was treated with 2400 cGy owing to a significant overlap of the target with the RVOT and proximity to great vessels.

### Clinical follow-up and survival after SBRT

SBRT was well tolerated in all patients. Given that patients were on telemetry throughout the SBRT procedure, device interrogation immediately after treatment confirmed that no VT episodes occurred during or after the procedure. Within the first 6 months after SBRT, 7 patients died after a median duration of 1.8 months (interquartile range 2.8) and 3 underwent cardiac transplantation for incessant VT (patient # 1, 15 days; patient # 2, 1 month; patient # 3, 3 months). Overall survival without undergoing a heart transplant was 60% at 6 months (Supplemental Figure 2). One patient was excluded from analysis owing to inadequate (n = 1) follow-up for VT burden. All deaths occurred after discharge and were not directly attributed to SBRT or its complications. Four patients had an active Do Not Resuscitate status and died of progressive heart failure, whereas the remaining 3 were hospitalized with severe dyspnea and died of mixed cardiogenic and septic shock.

### Primary endpoint

**Figure 5:**
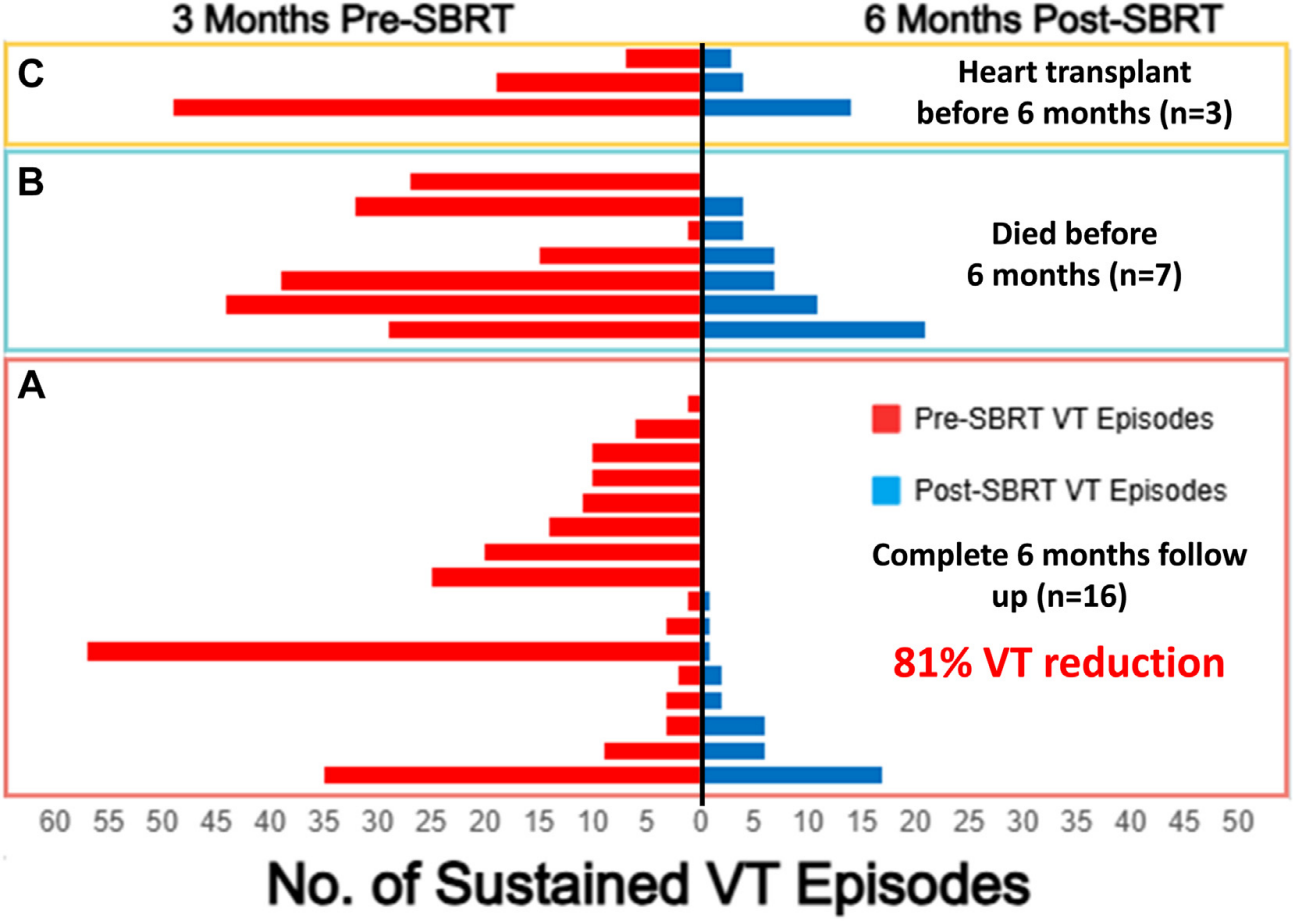
Reduction of VT burden after SBRT. Overall 25 patients underwent 27 individual SBRT sessions. **A:** Patients (n = 16) with a complete 6-month follow-up (lower panel). **B:** Patients (n = 7) who died <6 months after SBRT (middle panel). **C:** Patients (n = 3) who underwent cardiac transplant <6 months after SBRT (top panel). One patient with successful SBRT who is not displayed had only 3 months’ follow-up and did not have any episode of VT since treatment. Overall reduction of VT episodes was 81% for the 16 patients who survived a complete 6-month follow-up and had no transplant or further interventions for VT. SBRT = stereotactic body radiation therapy; VT = ventricular tachycardia.

**Figure 6:**
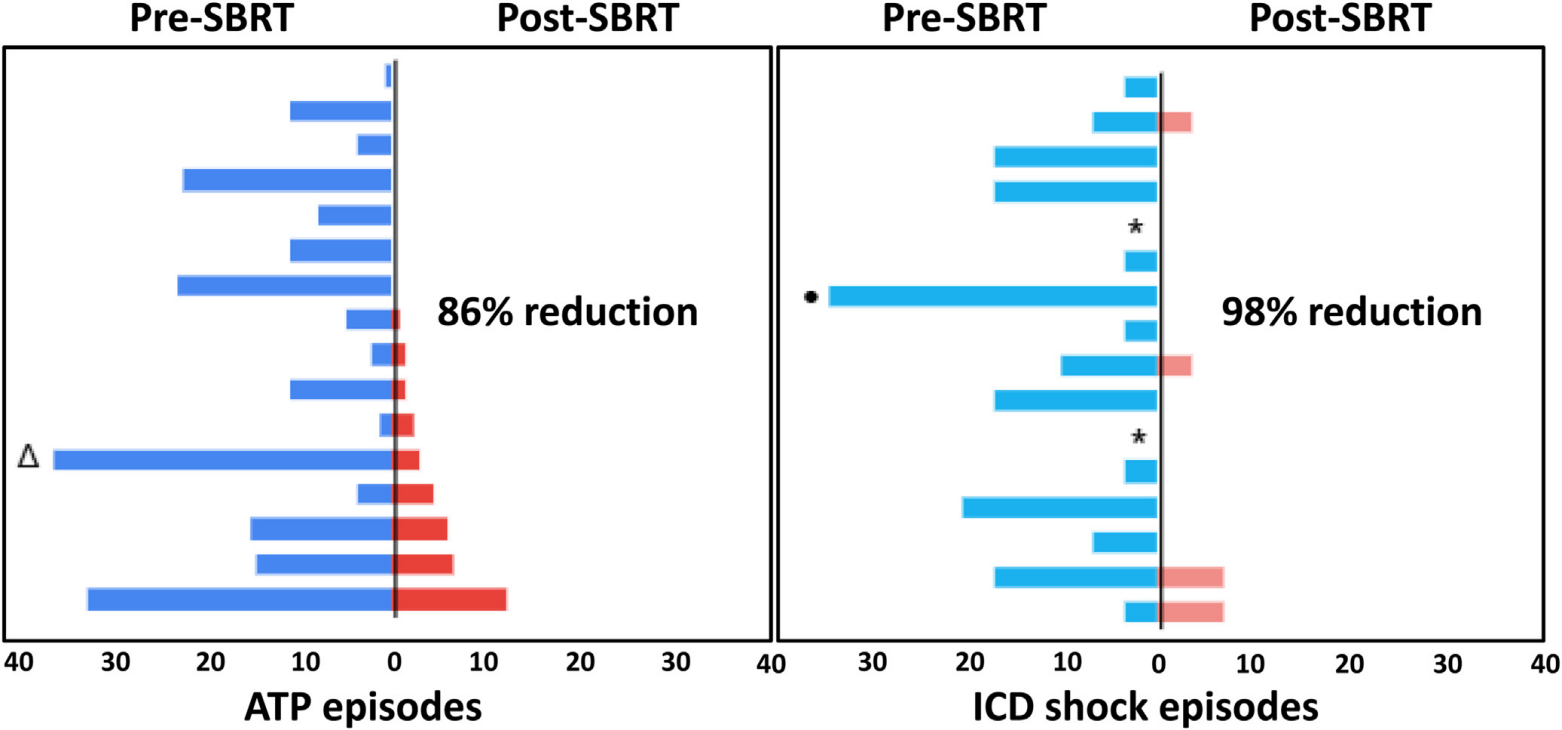
Reduction of ATP and ICD shock episodes after SBRT. For the 16 patients with a complete 6-month follow-up, mean % reduction in ATP and shock episodes per month was 86% (left panel) and 98% (right panel), respectively. ATP = antitachycardia pacing; ICD = implantable cardioverter-defibrillator; SBRT = stereotactic body radiation therapy.

Complete clinical follow-up for assessment of the primary endpoint of reduction of VT burden was available for 16 patients (16 of 27; 59%) whose cumulative reduction of VT burden per month was 81% (*P* < .05). Eight patients (50%) did not have any episode of VT during follow-up (Figure 5). Reduction of appropriate ATP and ICD shock per month was 86% and 98%, respectively (*P* < .05) (Figure 6). Among patients with a complete 6-month follow-up, the number of patients on at least 2 AAD decreased from 11 (69%) to 0 (0%) (*P* < .01) (Supplemental Figure 3).

### Complications during follow-up

Overall, SBRT was well tolerated without any acute issues during SBRT sessions. During short-term follow-up, the common side effects included nausea and vomiting (n = 7), fatigue (n = 5), and dizziness (n = 2). A more serious side effect that can be attributed to SBRT was diaphragmatic paralysis in 1 patient, in whom evaluation of dyspnea prompted imaging that revealed significant elevation of the left hemidiaphragm compared with a CT scan performed before SBRT. The SBRT target in this patient was basal and midlateral LV. Other significant side effects included the development of new pericardial effusion (n = 3) and pulmonary fibrosis (n = 1). Follow-up chest CT imaging was not performed routinely and was obtained based on clinical symptoms. Despite this, post-SBRT chest imaging (radiograph or CT) was available in most cases, with pulmonary fibrosis observed in only 1 patient. The patient who developed pulmonary fibrosis was also taking amiodarone for 6 months. Amiodarone was discontinued after the diagnosis of pulmonary fibrosis.

## Discussion

### Major findings

(1) Patients who survived for at least 6 months had a significant reduction (81%) in VT burden after SBRT. (2) Among those who underwent SBRT, 40% either died (n = 7) or underwent heart transplantation (n = 3) in the first 6 months, highlighting the overall morbidity in these patients. (3) SBRT may offer a viable option for patients ineligible for or who have failed previous CA. (4) SBRT target delineation requires a multidisciplinary effort using imaging and EP data.

### SBRT as an emerging technology

The role of SBRT for VT management is evolving.^1^^,^^10^ Although the outcomes are promising, there is significant selection bias and heterogeneity among patients undergoing SBRT. Typically, patients who were treated with SBRT have exhausted all existing options for VT management. Several of these patients developed complications from recurrent VT before SBRT. In our experience, there were 4 patients who had workup done but died before undergoing SBRT, which highlights the vulnerability and fragility of the population referred for SBRT. Among patients who survived the associated comorbidities and did not receive a transplant (Figure 3A), they demonstrated significant VT reduction (81% decrease). SBRT may offer benefit for heart failure patients with resistant VT; however, further research is needed to better define optimal patient selection and timing.

### Critical role of target delineation for SBRT

Adequate target delineation is critically important for SBRT. Besides the advantage of being a noninvasive treatment modality, SBRT should be viewed as a complementary strategy to CA by providing homogenous ablation in areas where CA is either ineffective (septal substrate) or unsafe (LV summit or outflow tract area). Existing VT targeting strategies incorporate EP (EKG, invasive mapping) and imaging data (scar identification) for the identification of the ablation target. EKG-based localization of VT has limitations.^16^ In clinical practice, the operators often rely on multiple EP characteristics obtained during invasive studies for the identification of the VT isthmus. The identification of such EP features requires tedious catheter-based mapping. Detailed mapping is often not feasible in patients with significant hemodynamic instability. Patients often referred for SBRT are not ideal for prolonged procedures needed for detailed mapping, compromising the quality of data obtained for target localization. In addition, a lack of a uniform platform/software to allow integration of EP data into the radiation planning system presents another challenge for the correct identification of the SBRT target. Although the 17-segment model served as a standardized framework for target delineation, it was not applied rigidly. In some cases, only subregions within a segment (eg, basal-inferior portion) were targeted based on the distribution of arrhythmogenic substrate and the proximity to adjacent critical organs. Full-segment coverage was avoided when not clinically justified, particularly when the burden of substrate was focal or confidence in the supporting data was low. Ventricular scar often harbors the critical area responsible for the initiation and maintenance of VT; therefore, imaging-based identification of scar plays a critical role in accurate SBRT target identification. In our experience, targets were typically located in the scar border zone or presumed VT exit sites, with localization guided by a combination of imaging, EP data, and device tracings (Table 2). Although cardiac MRI is the gold standard for the identification of ventricular scar, most patients who undergo target delineation for SBRT have defibrillators that, in certain regions, may significantly limit the availability of compatible MRI platforms and the quality of the images obtained. An optimal workup for SBRT target delineation should combine EP and imaging data. Until the accuracy of noninvasive mapping improves significantly, SBRT targeting may continue to require invasive mapping for the identification of VT sites. Although invasive EP studies were used in most patients to enable precise substrate mapping and confirm VT morphology via pace mapping, noninvasive programmed stimulation from ICDs may serve as a viable alternative. Further studies are needed to refine the workflow for SBRT target delineation. A multicenter, randomized trial comparing safety and efficacy between SBRT and repeat CA for patients with refractory VT is ongoing (NCT05765175).

### Limiting treatment-related toxicity

Safely delivering cardiac SBRT presents unique challenges for radiation oncologists, especially given the proximity of critical intrathoracic structures. Aside from the complexities of accurately defining and targeting the arrhythmogenic substrate, including the isthmus, careful attention was also given to minimizing radiation dose to adjacent intrathoracic organs. Although dose constraints were based on published thoracic and cardiac SBRT data, plan design also plays a central role. In our experience, we did not specifically prescribe an internal boost but also did not limit dose hotspots. This reflects a common radiosurgical planning strategy where enforcing strict homogeneity can worsen dose falloff and increase exposure to surrounding tissues.^17^ The approach of allowing the plan to arrive at an internal maximum dose that maintains sharp falloff has been used in early animal studies and the first human case and by other expert centers.^18^^,^^19^

#### Cardiac toxicity

Given that oncologic targets rarely overlap with the heart and the possibility that any radiation dose to the heart can affect cardiac function, the prescription dose for cardiac SBRT (2500 cGy in a single fraction) is significantly higher than cardiac dose radiation oncologists would ordinarily accept for oncologic intrathoracic targets (Radiation Therapy Oncology Group 0915). Although this dose is well tolerated in this experience, the authors recommend minimizing PTV size, if possible. As a matter of practice to avoid cardiac toxicity, the authors have avoided treatment in instances where PTV would be >200 mL and, when feasible, have endeavored to keep PTV at <150 mL.

#### Digestive organ toxicity

The stomach and esophagus can occasionally overlap with the target volume, particularly in cases where the inferior segments of the LV are being targeted. These luminal digestive organs are especially sensitive to radiation, and G3+ fistula and perforation have been reported by others after cardiac SBRT.^10^ To avoid these significant complications, the authors recommend selectively underdosing regions of the target that overlap significantly with these organs, restricting the maximum point dose to the stomach or esophagus to <1740 cGy.

#### Bronchial toxicity

Depending on patient anatomy and target location, central airways structures, such as the left mainstem bronchus, lingular bronchus, and left lower lobe bronchus, may approach the treatment field. To avoid significant complications such as pulmonary hemorrhage or stricture, the authors recommend selectively underdosing regions of the target that overlap significantly with these organs, restricting the maximum point dose to these organs to <2100 cGy.

#### Other mediastinal structures

In our institutional experience, 1 patient developed hemiparesis of their diaphragm, potentially owing to phrenic nerve toxicity. A retrospective review of the treatment plan demonstrated that a ∼3 cm segment of the phrenic nerve was within PTV expansion and exposed to 2550–2700 cGy, doses geometrically consistent with this location within the target. Although limiting hotspots may seem to be a safer strategy, in the “no hotspot limiting” approach, typically only the very central portion of the target exceeds ∼120% of the prescription dose, and even if hotspots were restricted, this dose would not be considered a “hotspot.” In this case, it is unlikely that restricting the hotspot would have reduced exposure to the phrenic nerve, and it may have actually increased high-dose exposure to adjacent tissues by worsening the dose gradient or R50 (V50%/target volume).^17^ There are only a few reported cases of phrenic nerve injury in the SBRT, and presently, its dose/volume tolerances are not well known.^20^ Although the authors do not recommend specifically contouring and/or avoiding the phrenic nerve, care should always be taken to minimize target size and maximize conformality to avoid unnecessary exposure of normal tissues.

### Starting a radiation ablation program

Before starting a cardiac radioablation program, appropriate expertise and technology are essential to ensure success and minimize toxicity. Patient selection should involve multidisciplinary discussion. Owing to the complexity of target selection, training with experienced centers is recommended. Institutions new to SBRT should collaborate with experienced teams for their initial cases.

## Conclusion

This study demonstrates the safety and efficacy of SBRT in a diverse patient population with refractory VT. Leveraging the experience of 27 cases, the authors describe a step-by-step workflow for accurate SBRT target delineation, a quintessential for successful outcome. The findings of this study support SBRT as a promising therapeutic option for patients unresponsive to conventional treatments, warranting further investigation in larger cohorts.

### Limitations

This study has several limitations. There could be a significant selection bias given that various centers have variable practices for the care of patients with recurrent VT. Although most patients in this report had at least 1 CA, some centers may pursue multiple CAs before considering SBRT. However, the patients who underwent SBRT after 1 CA were deemed inadequate candidates for repeat ablation for various patient- and procedure-related factors. Noninvasive programmed stimulation was not used in these cases for VT induction or target selection, given that it was not part of the standard workflow during the time period of this study. The retrospective and nonrandomized nature of the study is another limitation. Despite these limitations, this is the largest single-center SBRT experience with encouraging outcomes.
