# Supplementary material for: Target delineation workflow and outcomes of stereotactic cardiac radioablation
Source: Heart Rhythm O2. 2025 Jun 19;6(9):1401–11. doi: 10.1016/j.hroo.2025.06.009 (PMC12635712; doi:10.1016/j.hroo.2025.06.009)
Supplement: Supplemental Figures and Tables [file mmc1.docx]

**SUPPLEMENTARY DATA**

**TABLES (1-2)**

**Supplementary table 1: Anti-arrhythmic management of study population prior to SBRT**

| **Antiarrhythmic treatment modality** | **N=29 Patients** |
| --- | --- |
| **No. of prescribed antiarrhythmic drugs**     1     2  3 | 4 (14%)  20 (69%)  5 (17%) |
| **Pre-SBRT antiarrhythmic procedures**  Radiofrequency catheter ablation  Percutaneous stellate ganglion Block  Surgical Sympathectomy | 27 (93%)  5 (17%)  9 (31%) |
| **No. of previous catheter ablations**     0     1     2  3 | 2 (7%)  3 (10%)  15 (52%)  9 (31%) |

**Supplementary table 2: Characteristics of target area in cases treated with SBRT (N=27)**

| **Planning Treatment Volume (**cm^3^**)** | 131.3 ± 34.8 |
| --- | --- |
| **SBRT Target location**  Left Ventricle  Outflow Tract  Periaortic Area  RV Outflow | 17 (63%)  10 (37%)  9 (33%)  1 (4%) |
| **Number of Segments on the 17-segment model**  1  2  3  4  5 | 1 (4%)  11 (40%)  7 (26%)  7 (26%)  1 (4%) |

**Supplementary Figures Legend (1-3)**

**Supplementary Figure 1:**

**
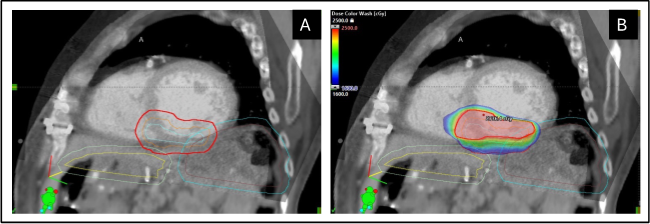
**

**Selective underdosing of cardiac SBRT target when adjacent to sensitive digestive organs:** Representative case where the target in the inferior wall of LV could not safely be treated to full prescription dose due to the presence of the stomach and large bowel immediately subjacent to the diaphragm.  **(A)** Short axis view of the left and right ventricle where the clinical target volume (CTV) is shown in cyan color, the iterative target volume (ITV) in orange and planning treatment volume (PTV) in red. The PTV is approaching the stomach (yellow) and large bowl (brown) overlapping significantly with their respective planning at risk volume (PRV). **(B)** Given that prescribing 2500 cGy (standard cardiac dose) to cardiac SBRT target could result in significant toxicity to these luminal GI structures, the regions of overlap were selectively underdosed. In the treatment plan, maximum dose to the stomach and stomach-PRV were 1000 cGy and 1800 cGy, respectively, and maximum dose to large bowel and large bowel_PRV were 2500 cGy and 2700 cGy, respectively.

**Supplementary Figure 2:**

**
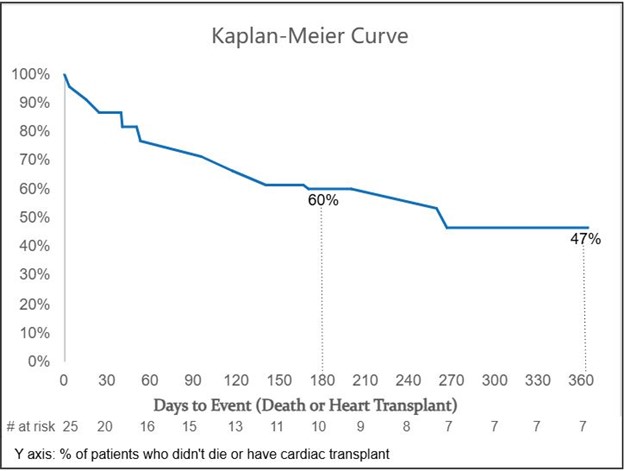
**

**Kaplan Meir Time-to-Event Analysis**: The Kaplan Meir curve shows time to death or cardiac transplant. At 6 months post-SBRT, 7 patients died and 3 underwent cardiac transplant. Overall survival at 6 and 12 months was 60% and 47%, respectively.

**Supplementary Figure 3:**

**
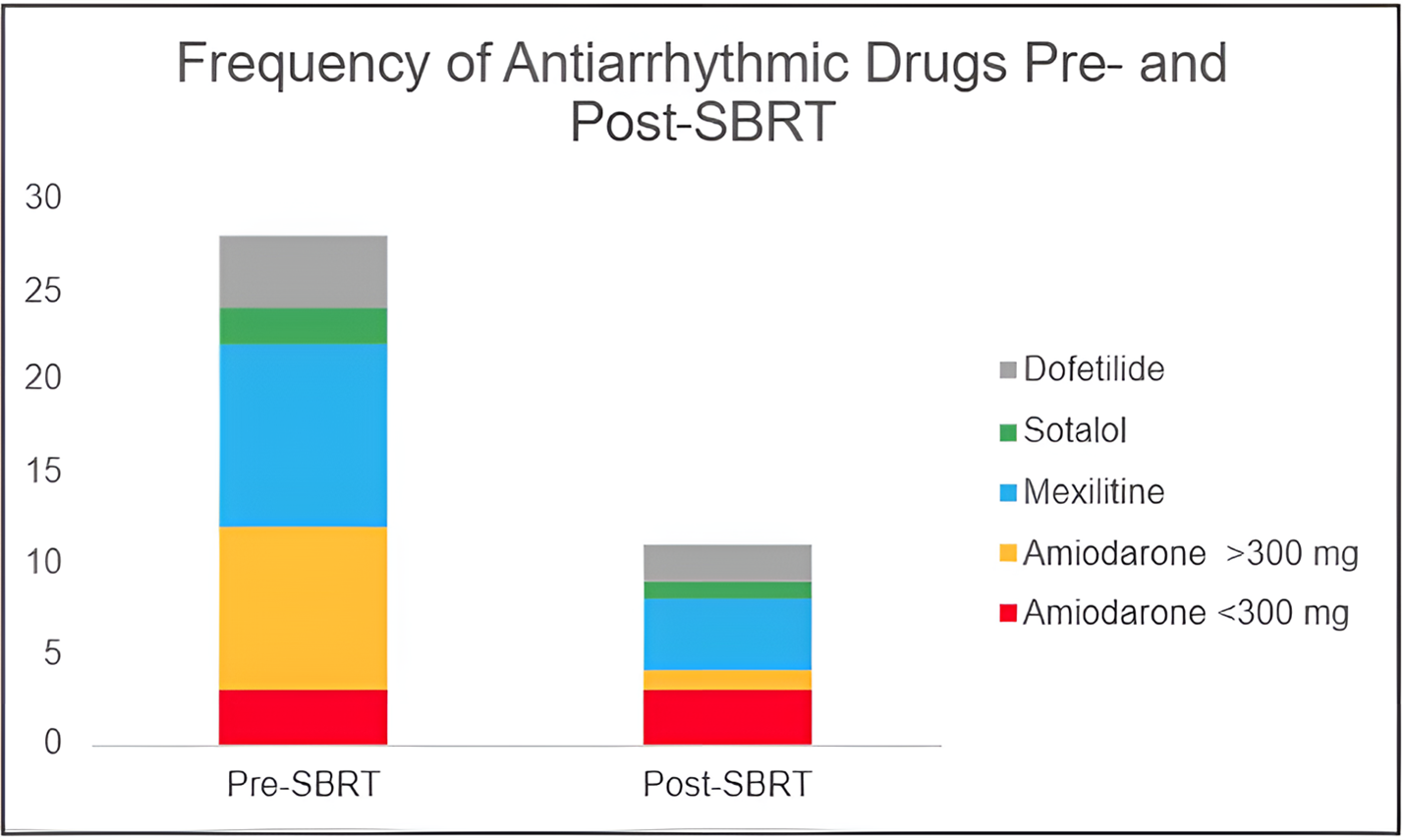
**

**Antiarrhythmic Drugs Pre- and Post-SBRT**: For patients with complete 6 months follow up (n=16), the number of patients on at least 2 antiarrhythmic medications decreased from 11 (69%) to 0 (0%), [P<0.01].
